# Combination therapy with cefiderocol and minocycline for *Stenotrophomonas maltophilia* bacteremia in an obese patient on maintenance hemodialysis: A case report

**DOI:** 10.1016/j.idcr.2026.e02742

**Published:** 2026-08-31

**Authors:** Kosuke Kameda, Keisuke Sawada, Kuniaki Sano, Nobuyuki Suzuki, Natsue Honda, Kosuke Haruki

**Affiliations:** a Department of Pharmacy, Dokkyo Medical University Saitama Medical Center, 2-1-50 Minami-Koshigaya, Koshigaya, Saitama, 343-8555, Japan; b Division of Infection Control, Dokkyo Medical University Saitama Medical Center, 2-1-50 Minami-Koshigaya, Koshigaya, Saitama, 343-8555, Japan; c Department of Pharmacy, Federation of National Public Service Personnel Mutual Aid Associations Hirakata Kohsai Hospital, 1-2-1 Fujisaka-higashi-machi, Hirakata-shi, Osaka, 573-0153, Japan

**Keywords:** *Stenotrophomonas maltophilia*, Cefiderocol, Minocycline, Obesity, Maintenance hemodialysis, Case report

## Abstract

*Stenotrophomonas maltophilia* bacteremia is associated with high mortality and multidrug resistance and has limited treatment options. Clinical evidence for cefiderocol (CFDC) and combination regimens remains scarce. Herein, we report a case of *S. maltophilia* bacteremia, which emerged following initial polymicrobial bloodstream, intra-abdominal, and pleural infections, successfully treated with a combination of CFDC and minocycline (MINO) in a man in his 40s. Faced with constraints such as obesity (body mass index, 35 kg/m^2^), maintenance hemodialysis, and a temporary CFDC shortage, the dosage was optimized stepwise based on pharmacokinetic principles and the dialysis modality, alongside appropriate source control. Bacterial clearance was achieved without recurrence. This case adds valuable clinical insight to the literature by suggesting that combination therapy with CFDC and MINO may serve as a feasible, safe, and effective therapeutic option for *S. maltophilia* bacteremia in obese patients undergoing maintenance hemodialysis. Additionally, it provides a practical example of a rational, pharmacokinetic/pharmacodynamic-based decision-making process under multiple concurrent constraints, including drug shortages and uncertain antimicrobial susceptibility. However, given the inherent limitations of a single case report, prospective pharmacokinetic studies and comparative clinical data are needed before generalized recommendations can be made.

## Highlights

• Combined cefiderocol-minocycline contributed to clearing *S. maltophilia* bacteremia.
• Obesity, dialysis, and drug shortages compounded the dosing challenge in this case.
• Antimicrobial decision pathway under multiple constraints is detailed step by step.
• Cefiderocol’s PK profile was considered favorable across the CHD-to-IHD transition.
• Prospective PK/PD studies remain needed before the regimen can be generalized.

## Introduction

*Stenotrophomonas maltophilia* is an important opportunistic pathogen that frequently exhibits multidrug resistance, resulting in a high reported mortality rate (14–69%) for bacteremia, particularly among immunocompromised hosts [1,2]. Effective therapeutic options remain limited as the current conventional antimicrobial agents, such as trimethoprim-sulfamethoxazole (TMP-SMX), levofloxacin (LVFX), and minocycline (MINO), show only limited success [2]. Although dual-agent combination therapy is recommended for severe cases and immunocompromised hosts, only a few large-scale clinical trials in which individual agents are compared with each other or with combination regimens in terms of efficacy have been conducted [2]. Further challenges complicating the management of *S. maltophilia* infections include the scarcity of pharmacokinetic/pharmacodynamic (PK/PD) data to substantiate optimal dosing regimens, as well as the poor correlation between minimum inhibitory concentrations (MICs) and clinical outcomes [2,3].

Cefiderocol (CFDC) is a siderophore cephalosporin first approved by the US Food and Drug Administration (FDA) in 2019 for complicated urinary tract infections, including pyelonephritis, in adults with limited or no alternative treatment options. Subsequently, in 2020, the European Medicines Agency (EMA) granted it a broader approval for the treatment of infections caused by aerobic Gram-negative organisms in adults with limited treatment options. It was later approved in Japan in 2023. It exhibits activity against multidrug-resistant Gram-negative bacteria, including *S. maltophilia* [4]. There is a paucity of clinical evidence of the efficacy of CFDC for *S. maltophilia* bacteremia, with a series of eight cases reported by Vena et al. representing the largest study to date [5]. Furthermore, although combination therapy with CFDC and MINO has been proposed as a therapeutic strategy [1,6], current evidence is limited to *in vitro* data. Additionally, only a few instances of employing CFDC in patients with complex clinical profiles, such as those with obesity who are undergoing dialysis, have been reported worldwide [5,[7], [8], [9]].

Herein, we describe a case of *S. maltophilia* bacteremia managed with a combination of CFDC and MINO, alongside appropriate source control, in an obese patient undergoing maintenance hemodialysis (HD). We report this clinical experience to illustrate the rationale behind the antimicrobial decision-making process required when patients are treated for this infection under multiple constraints, including host complexity, drug shortages, and challenges in applying revised MINO breakpoints.

## Case report

A man in his 40s with a body mass index (BMI) of 35 kg/m^2^, chronic kidney disease, and type 2 diabetes mellitus experienced several days of chest pain. He was transferred to our hospital after exhibiting fever and pleural effusion at his regular dialysis clinic.

**Fig. 1:**
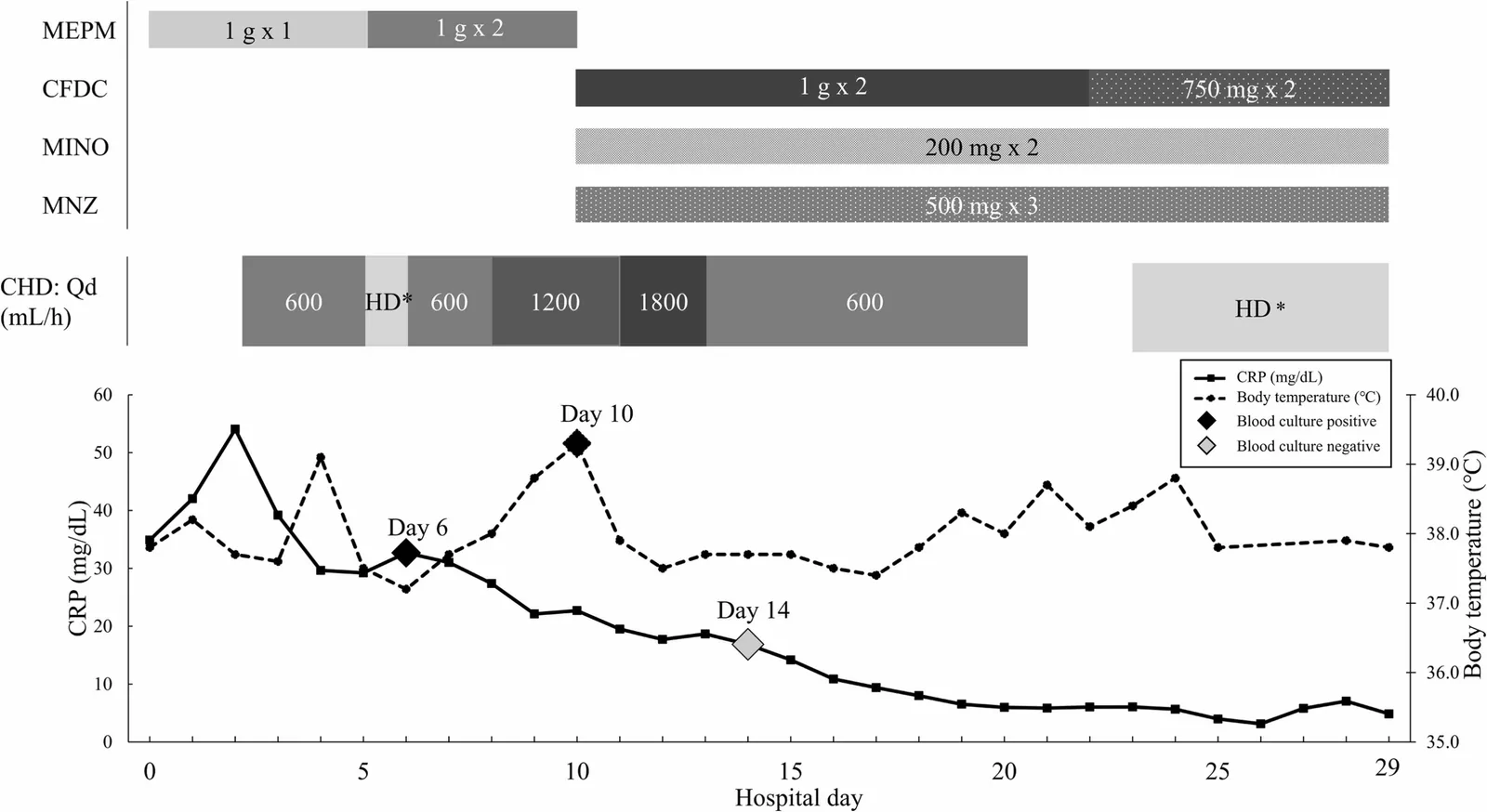
Clinical course after admission. The upper panel shows the antimicrobial regimens administered during hospitalization. MEPM was initiated at 1 g once daily and increased to 1 g twice daily. After identification of *S. maltophilia* on Day 10, combination therapy with CFDC, MINO, and MNZ was started. CFDC was administered at 1 g twice daily during CHD (a restricted dose due to limited stock availability) and adjusted to 750 mg twice daily upon transition to intermittent HD on Day 22. The middle panel depicts the dialysis modality and dialysate flow rate (Qd) during CHD. The lower panel shows CRP levels (solid line, left axis) and body temperature (dashed line, right axis). Black diamonds indicate positive blood cultures (Day 6 and 10); gray diamonds indicate negative blood cultures confirming bacterial clearance (Day 14). * HD settings: Blood flow rate, 150–180 mL/min; Duration, 4 h. Abbreviations: MEPM, meropenem; CFDC, cefiderocol; MINO, minocycline; MNZ, metronidazole; CHD, continuous hemodialysis; Qd, dialysate flow rate; HD, hemodialysis; CRP, C-reactive protein.

Upon arrival, his vital signs included a Glasgow Coma Scale score of 15, respiratory rate of 30 breaths/min, heart rate of 110 beats/min, blood pressure of 92/50 mmHg, oxygen saturation of 97% on a 2-L/min nasal cannula, and body temperature of 39.0°C. These findings yielded a quick Sequential Organ Failure Assessment score of 2, indicating a risk of clinical deterioration. Based on a comprehensive assessment combining this score with his overall severe clinical presentation, sepsis was strongly suspected. Subsequent computed tomography (CT) demonstrated free air in the ileocecal region and right retroperitoneum. Given the suspicion of an intra-abdominal infection secondary to gastrointestinal perforation, an emergency laparotomy and peritoneal lavage were performed on the same day. Laboratory findings at admission (Table 1) further corroborated the diagnosis of sepsis, leading to the patient’s postoperative admission to the intensive care unit (ICU) (Fig. 1).

**Table 1:** Laboratory findings at admission.

**Parameter**	**Value**
**Hematology**	
WBC	13,200 /μL
Hb	14.6 g/dL
PLT	134 × 10 ³ /μL
**Biochemistry**	
CRP	44.8 mg/dL
T-bil	1.26 mg/dL
AST	133 U/L
ALT	55 U/L
BUN	83 mg/dL
Cr	11.7 mg/dL
Alb	2.3 g/dL
Na	128 mEq/L
K	5.9 mEq/L
**Coagulation & Blood gas**	
PT-INR	1.00
pH	7.34
PaO2	54.9 mmHg
Lactate	27.8 mg/dL

**Abbreviations: Alb**, albumin; **ALT**, alanine aminotransferase; **AST**, aspartate aminotransferase; **BUN**, blood urea nitrogen; **Cr**, creatinine; **CRP**, C-reactive protein; **Hb**, hemoglobin; **K**, potassium; **Na**, sodium; **PaO2**, partial pressure of arterial oxygen; **PLT**, platelet; **PT-INR**, prothrombin time-international normalized ratio; **T-bil**, total bilirubin; **WBC**, white blood cell.

Antimicrobial therapy was initiated perioperatively with meropenem (MEPM) at a dose of 1 g once daily. *Escherichia coli* and *Clostridium ramosum* were isolated from admission blood cultures. Additionally, *E. coli*, *Klebsiella pneumoniae*, *Bacteroides thetaiotaomicron*, and *Streptococcus anginosus* were detected in the culture grown from ascites obtained during the surgery, prompting the continuation of MEPM therapy. On day 2, an emergency vascular access catheter was inserted, and continuous HD (CHD) was initiated. On day 6, owing to a persistent fever, additional blood was obtained for culturing. CT revealed empyema; consequently, a chest tube was inserted. Cultures grown from the pleural fluid yielded *E. coli* and *Bacteroides fragilis*. At the same time, the MEPM dosage was increased to 1 g twice daily, considering the patient’s BMI.

On day 10, two sets of blood cultures obtained on day 6 yielded *S. maltophilia*. Repeat blood cultures collected on day 10, just prior to initiation of the new antimicrobial regimen, also subsequently yielded *S. maltophilia*. The antimicrobial regimen was changed to CFDC (1 g twice daily) and MINO (200 mg twice daily) while requesting susceptibility testing for CFDC. Furthermore, to cover the *C. ramosum* isolated from the admission blood cultures, we added metronidazole (MNZ) at a dose of 500 mg three times daily. On day 11, the antimicrobial susceptibility profile of the *S. maltophilia* became available (Table 2). The isolate exhibited susceptibility to CFDC based on Clinical and Laboratory Standards Institute (CLSI) criteria, assessed via the disk diffusion method by using KB Disk Eiken Cefiderocol disks (Eiken Chemical Co., Ltd.); the inhibition zone was 30 mm. However, the minimum inhibitory concentration (MIC) was not quantified because the broth microdilution method was unavailable at our facility. Furthermore, the isolate was susceptible to MINO (MIC ≤2 μg/mL) according to the breakpoints established in the 33rd edition of the CLSI M100 document (before the 2024 revision).

**Table 2:** Antimicrobial susceptibility profile of *Stenotrophomonas maltophilia* isolated from blood cultures on day 6.

**Tested antibiotics**	**MIC (μg/mL)**	**Susceptibility**
Ceftazidime	> 16	R
Levofloxacin	1	S
Minocycline	≤ 2	S^a^
Trimethoprim/sulfamethoxazole	≤ 2/38	S
Cefiderocol	-	S^b^

S: susceptible, R: resistant, ^a^ At the time of treatment, the minocycline MIC (≤ 2 μg/mL) was interpreted as susceptible under the previous CLSI M100 breakpoints (S ≤ 4 μg/mL; 33rd edition). However, since the 34th edition (2024), the *S. maltophilia*–specific breakpoints were revised to S ≤ 1, I = 2, R ≥ 4 μg/mL [10]; because the reported MIC of ≤ 2 μg/mL could represent a true value of either ≤ 1 (susceptible) or 2 (intermediate), susceptibility cannot be confirmed under the current criteria. ^b^ Zone diameter: 30 mm (disk diffusion method).The MIC was not quantified because the broth microdilution method was unavailable at our facility.

On day 14, follow-up blood cultures confirmed bacterial clearance. On day 15, the catheters were exchanged; however, the catheter tips were not submitted for culturing. On day 22, the dialysis modality was transitioned from CHD to intermittent HD. Consequently, we adjusted the CFDC dosage to 750 mg twice daily. We continued combination treatment for 15 days from the first negative blood culture, in accordance with catheter-related bloodstream infection (CRBSI) guidelines.

On day 29, the CFDC–MINO–MNZ combination therapy was completed. By the end of this regimen, the patient’s clinical condition and respiratory status had significantly improved, allowing discontinuation of supplemental oxygen. Other inflammatory markers (white blood cell count [WBC], 8500 /μL) and liver function parameters (aspartate aminotransferase, 34 U/L; alanine aminotransferase, 24 U/L) had improved compared to admission. Total bilirubin (1.94 mg/dL) was steadily recovering from an in-hospital peak of 12.55 mg/dL, fully normalizing by day 33. No adverse events attributable to the combination therapy were observed.

The antimicrobial regimen was de-escalated to intravenous sulbactam/ampicillin (3 g once daily; days 30–40), targeting the pathogens previously isolated from the blood (*E. coli* and *C. ramosum*) and pleural fluid (*E. coli* and *B. fragilis*). Following a brief pause for clinical evaluation (days 41–44), sulbactam/ampicillin was resumed (days 45–47) due to persistent drainage from the chest tube, and was subsequently switched to oral amoxicillin/clavulanate (250 mg once daily; days 48–52) for continued empyema management. Later, the patient developed a new fever, transient leukocytosis, and elevated C-reactive protein (CRP) levels with CT-confirmed pneumonia, prompting the administration of LVFX (250 mg every other day; days 53–64) targeting both the respiratory infection and the residual empyema.

During the clinical course, routine and fever-workup cultures obtained from pleural fluid, ascites, and sputum yielded various other microorganisms (e.g., *Enterococcus faecalis* and *Candida* species). However, in the absence of corresponding infectious signs, these isolates were clinically interpreted as colonizers, and targeted antimicrobial therapy was deemed unnecessary. Importantly, follow-up blood cultures obtained on days 44 and 55 remained negative, confirming no recurrence of *S. maltophilia* bacteremia.

Follow-up CT scans on days 35, 53, and 60 showed a gradual improvement of the empyema, which was managed with continued drainage until all drains were sequentially removed by day 60. All antimicrobial therapies were successfully discontinued on day 64. Furthermore, no adverse events associated with any of the subsequent antimicrobial regimens were observed. By the end of antimicrobial therapy, the WBC and all other inflammatory markers, except for CRP, had fully normalized. CRP levels plateaued at approximately 5 mg/dL until discharge, which we attributed to the residual empyema. A final CT scan on day 73 confirmed that the residual empyema cavity remained stable. Following the completion of antimicrobial therapy, intensive physical rehabilitation and nutritional management were continued to treat disuse syndrome and deconditioning resulting from the prolonged illness. After achieving full physical independence through rehabilitation, the patient was successfully discharged on day 100 and has not required readmission.

## Discussion

In this highly complex case characterized by obesity, dialysis, and restricted drug availability, we successfully managed *S. maltophilia* bacteremia by using a tailored, PK/PD-based antimicrobial strategy with CFDC and MINO. Although the absence of new infiltrates on imaging and fluid cultures negative for *S. maltophilia* raised suspicion for a CRBSI, bacterial translocation from the preceding gastrointestinal perforation could not be definitively excluded. Consequently, the infection was managed as a suspected CRBSI with appropriate source control (catheter replacement) and antimicrobial therapy [11].

Given the patient’s critical condition (postoperative sepsis and dialysis-dependent kidney disease) and gastrointestinal unavailability, intravenous dual-agent therapy was indicated [2]. We evaluated four agents: TMP-SMX, LVFX, MINO, and CFDC. Because established PK/PD guidelines for patients with concurrent obesity and dialysis are lacking, regimen construction required theoretical reasoning based on each agent’s PK profile.

First, TMP-SMX was excluded. In critically ill patients who require kidney replacement therapy, TMP-SMX treatment carries high risks of clinical (60%) and microbiological (86%) failure [12]. Additionally, the substantial diluent volume required raised concerns of volume overload [13].

Second, LVFX has a low susceptibility rate (65%) in Japan [14]. Furthermore, among ICU patients, PK/PD targets may not be reached depending on the MIC [3]. Our isolate demonstrated an MIC of 1 μg/mL, at which the standard high-dose regimen (750 mg once daily) yields a probability of target attainment (PTA) of only 72.2% [3].

Third, MINO was considered. Because it is hepatically metabolized, kidney-related dosage adjustments are unnecessary [15]. Its high lipophilicity increases the volume of distribution in obesity, necessitating an escalated dosage (200 mg twice daily), which achieves adequate PK/PD targets if the MIC is ≤ 1 μg/mL [3]. Although our isolate (MIC ≤2 μg/mL) was susceptible under previous CLSI criteria (S for MIC ≤4 μg/mL), the 2024 revised breakpoints (S for MIC ≤1 μg/mL, I for MIC = 2 μg/mL, and R for MIC ≥4 μg/mL) [10] rendered its susceptibility uncertain. Nevertheless, relying on reported *in vitro* synergy between MINO and CFDC [6], we reasoned that this combination would provide complementary activity to overcome this limitation.

Finally, CFDC was selected as the backbone therapy. As a hydrophilic agent that is primarily eliminated by the kidneys, body weight minimally influences its PK profile, obviating the need for dose adjustments for obese patients [4,16,17]. Furthermore, dosing regimens for CFDC during kidney replacement therapies have been established [4]. Given its favorable susceptibility profile [2], it represented a viable empirical option. However, because routine stocking of CFDC is rare in Japan, securing an adequate emergency supply proved challenging. Consequently, its dosing was optimized stepwise. During initial CHD, limited stock necessitated a restricted dose of 1 g twice daily rather than the recommended 1.5 g twice daily [4,18]. In the absence of direct evidence for such a modification, we used the continuous hemofiltration dosage as an alternative benchmark, while acknowledging that the modalities are not strictly equivalent. Upon transitioning to intermittent HD, the dosage was adjusted to the recommended 750 mg twice daily [4], meticulously timed to yield a drug clearance of approximately 60% per session.

Although de-escalation to monotherapy was considered after susceptibility results were obtained, we maintained the combination regimen for five reasons. First, MINO monotherapy was unfeasible owing to uncertain susceptibility under the revised CLSI criteria. Second, CFDC monotherapy was avoided during CHD because limited drug availability necessitated a restricted dose, requiring the complementary effects of MINO. Third, dual-agent therapy is recommended for severe infections even after CFDC dose optimization [2]. Fourth, CFDC and MINO reportedly exhibit *in vitro* synergy against multidrug-resistant *S. maltophilia* [6]. Fifth, clinical evidence was insufficient to justify the safety of switching to monotherapy for this critically ill patient. We prioritized his clinical safety to minimize the risk of therapeutic failure.

Notably, bacteremia had persisted despite preceding source control measures in this case, as confirmed by repeat positive cultures from blood drawn immediately prior to initiation of the new regimen (day 10). However, bacterial clearance was confirmed by the first follow-up blood culture on day 14—after combination therapy initiation and prior to catheter exchange (day 15). Although causation cannot be established from a single case, this chronological sequence suggests that the combination therapy contributed to resolving the infection.

Previous reports on CFDC for *S. maltophilia* bacteremia primarily described monotherapy or combinations with TMP-SMX or LVFX [5,[7], [8], [9],^19^] (Table 3). Few details of the practical decision-making process for CFDC and MINO treatment under concurrent constraints, such as obesity, dialysis, drug shortages, and uncertain susceptibility, have been reported. Sharing our stepwise approach provides a practical reference for similar complex scenarios in future.

**Table 3:** Summary of Published Cases of *Stenotrophomonas maltophilia* Bacteremia Treated with Cefiderocol.

**Case**	**Reference**	**Age / Sex**	**Underlying condition**	**RRT**	**Source of infection**	**CFDC dosage**	**Concomitant agent**	**Outcome**
1	Vena et al [5].	47 / F	Viral myocarditis, HLH	N/A^a^	CRBSI	2 g q8h	LVFX, TMP-SMX	Survived
2	Vena et al [5].	82 / F	ALL, breast cancer	N/A^a^	CRBSI	2 g q8h	None	Survived
3	Vena et al [5].	21 / M	90% TBSA burns	N/A^a^	Wound infection / BSI	2 g q8h	TMP-SMX	Died^b^
4	Vena et al [5].	18 / M	Lymphoma, aHSCT	N/A^a^	CRBSI	2 g q8h	None	Died^c^
5	Lupia et al [7]^. d^	N/A	HM	N/A^a^	NP with BSI	N/A	None	Survived
6	Lupia et al [7]^. d^	N/A	HM	N/A^a^	CRBSI	N/A	TMP-SMX	Survived
7	Medioli et al [8]^. d^	62 / F	Cardiomyopathy, CKD, DM	Yes^e^	CRBSI (septic thrombosis)	0.75 g BID	LVFX, TMP-SMX	Survived
8	Fratoni et al [9]^. d^	58 / M	Cardiomyopathy, shock	Yes^e^	Polymicrobial BSI & NP	2 g q12h	TGC	Died^b^
9	Hsu et al [19].	5w / M	TGA, ECMO	No	Persistent BSI	40 mg/kg q8h	None	Survived
**This** **case**	**Present case**	**40s / M**	**Obesity, CKD, DM**	**Yes**^**e**^	**BSI**	**1 g BID** **→ 750 mg BID**	**MINO, MNZ**	**Survived**

**Abbreviations:** aHSCT, allogeneic hematopoietic stem cell transplantation; ALL, acute lymphoblastic leukemia; BID, twice daily; BSI, bloodstream infection; CFDC, cefiderocol; CHD, continuous hemodialysis; CKD, chronic kidney disease; CRBSI, catheter-related bloodstream infection; CVVHDF, continuous venovenous haemodiafiltration; DM, diabetes mellitus; ECMO, extracorporeal membrane oxygenation; F, female; HD, hemodialysis; HLH, hemophagocytic lymphohistiocytosis; HM, hematological malignancies; IHD, intermittent hemodialysis; LVFX, levofloxacin; M, male; MINO, minocycline; MNZ, metronidazole; N/A, not available / not detailed in the text; NP, nosocomial pneumonia; RRT, renal replacement therapy; TBSA, total body surface area; TGA, transposition of great arteries; TGC, tigecycline; TMP-SMX, trimethoprim-sulfamethoxazole. ^a^ A third RRT case was reported in the aggregate data of Vena et al. [5], but individual case details were not available. ^b^ Microbiological clearance of *S. maltophilia* was achieved, but the patient ultimately died due to the severity of underlying conditions. ^c^ Patient died on the 4th day of treatment; follow-up blood cultures were not performed (clinical failure). ^d^ These previously published cases were also included in the eight-case series reviewed by Vena et al. [5]. ^e^ Specific RRT modalities: Case 7, HD; Case 8, CVVHDF; This case, CHD (Day 2–21) transitioned to IHD (Day 22 +)

This report has several limitations. First, the lack of measured antimicrobial blood concentrations precluded a quantitative assessment of PK/PD target attainment [20]. Second, the *in vivo* synergistic effect of MINO [6] could not be objectively verified. Third, isolating the precise contribution of each concurrent intervention (e.g., antimicrobials, source control, and dialysis alterations) to the patient’s recovery remains challenging. Fourth, because the broth microdilution method was unavailable, CFDC susceptibility was assessed solely via disk diffusion, leaving the precise MIC undetermined. Fifth, procalcitonin was not routinely measured per institutional protocols. Consequently, the therapy duration was comprehensively determined based on clinical resolution, normalization of inflammatory markers, radiological improvement, and microbiological clearance, rather than procalcitonin-guided decision-making. Finally, this was a single-center, single-case report.

In conclusion, this case demonstrates a rational, PK/PD-based decision-making process for the combined use of CFDC and MINO under multiple constraints, including obesity, dialysis, drug shortages, and uncertain antimicrobial susceptibility. Owing to the successful clinical outcome, we report our strategy of combination therapy, in conjunction with appropriate source control, as a practical example of constructing a rational therapeutic strategy tailored to the unique dosing requirements of obese patients undergoing dialysis. However, prospective PK studies and comparative effectiveness data are needed before generalized recommendations can be made.

## Author contributions

All the authors meet the ICMJE authorship criteria. All authors have read and approved the final version of the manuscript.

## CRediT authorship contribution statement

**Kosuke Kameda:** Writing – original draft, Conceptualization. **Keisuke Sawada:** Writing – review & editing, Writing – original draft, Conceptualization. **Kuniaki Sano:** Writing – review & editing, Conceptualization. **Nobuyuki Suzuki:** Writing – review & editing. **Natsue Honda:** Writing – review & editing. **Kosuke Haruki:** Writing – review & editing, Supervision.

## Patient consent

This case report was reviewed and approved by the Ethics Committee of Dokkyo Medical University Saitama Medical Center (approval no. 25129). Written informed consent was obtained from the patient for the publication of this case report.

## Ethics declaration

Written informed consent to take part in the study and to publish the article has been obtained from all participants or their legal representatives. The privacy rights of participants have been observed.

This study was performed in compliance with relevant laws, regulatory frameworks and guidelines where the research took place. This study was approved by the the Institutional Review Board (or Ethics Committee) of Dokkyo Medical University Saitama Medical Center. (Approval No. 25129)

This research follows the CARE guidelines and the CARE checklist.

## Funding

This report did not receive any specific grant from funding agencies in the public, commercial, or not-for-profit sectors.

## Declaration of Generative AI and AI-assisted technologies in the writing process

During the preparation of this work the authors used Gemini 3.1 Pro (Google) in order to refine and improve English language expressions. After using this tool, the authors reviewed and edited the content as needed and take full responsibility for the content of the published article.

## Declaration of Competing Interest

The authors declare that they have no known competing financial interests or personal relationships that could have appeared to influence the work reported in this paper.
